# Evaluation of a Slipped Capital Femoral Epiphysis Virtual Reality Surgical Simulation for the Orthopaedic Trainee

**DOI:** 10.5435/JAAOSGlobal-D-22-00028

**Published:** 2022-04-25

**Authors:** Adam Margalit, Krishna V. Suresh, Majd Marrache, Jonathon M. Lentz, Rushyuan Lee, John Tis, Ranjit Varghese, Brooke Hayashi, Amit Jain, Dawn Laporte

**Affiliations:** From the Department of Orthopaedic Surgery, Johns Hopkins University School of Medicine, Baltimore, MD.

## Abstract

The purpose of this study was to compare outcomes between orthopaedic trainees using various preoperative training platforms (physical simulation [PS], virtual reality [VR], and reading/videos) in a slipped capital femoral epiphysis model.

**Methods:**

Participants were randomly assigned to one of the three groups: (1) reading/video control group (n = 7), (2) VR group (n = 7), or (3) PS group (n = 7). Participants in the VR group completed a VR slipped capital femoral epiphysis module while participants in the PS group practiced the placement of a screw in the physical module before evaluation of percutaneous screw placement in the PS model. Outcomes evaluated included overall surgical time, amount of fluoroscopy, Global Rating Scale score, radiographic screw position, physical screw accuracy, presence of breeching of the articular surface or femoral neck, and overall platform rating (0 to 10).

**Results:**

No difference was observed in surgical time, Global Rating Scale score, radiographic or physical accuracy of screw position, or articular surface breaching between the groups. Subjectively, there was a difference in utility of platform rating between the groups (PS: 10 ± 0, VR: 7 ± 2, and control: 6 ± 1, *P* = 0.001).

**Conclusion:**

Training with VR was subjectively rated higher in value compared with reading/video methods and had similar performance outcomes compared with training with PS.

Surgical skills simulation allows for the practice of procedures in a risk-free environment and has been shown to improve patient safety and optimize clinical outcomes. Although not widely adopted, both traditional virtual reality (VR) surgical platforms (plastic models with arthroscopic haptic and tactile feedback with interactive surgical instruments) and more modern VR systems (simulators with head-mounted display and generic hand controllers) may have some benefit in orthopaedic training. Previous literature reports that VR simulator platforms may improve resident skill level in the operating room or on cadaver models for knee arthroscopy or total hip arthroplasty.^1,2,3,4,5,6^ Other studies have demonstrated improvements in procedural accuracy and rate of successful completion of tibial intramedullary nailing among medical students trained with VR headsets compared with a technique guide review.^7-9^ However, there is a paucity of literature that has directly compared VR as a surgical training platform in pediatric orthopaedic procedures with other educational platforms in novice orthopaedic trainees, with limited information on the transferability of acquired skills from simulation to the operating room.

Slipped capital femoral epiphysis (SCFE) screw fixation is a routine pediatric orthopaedic procedure which demands advanced three-dimensional skills. The utility of VR as a surgical learning platform to train novice orthopaedic trainees on how to perform in situ screw fixation has not yet been evaluated. The primary purpose of this study was to compare screw placement accuracy and surgical technique among orthopaedic trainees trained with either VR, isolated text, video, and literature review or physical simulation (PS) training in a SCFE sawbones model.

## Methods

### Study Design and Participants

**Figure 1:**
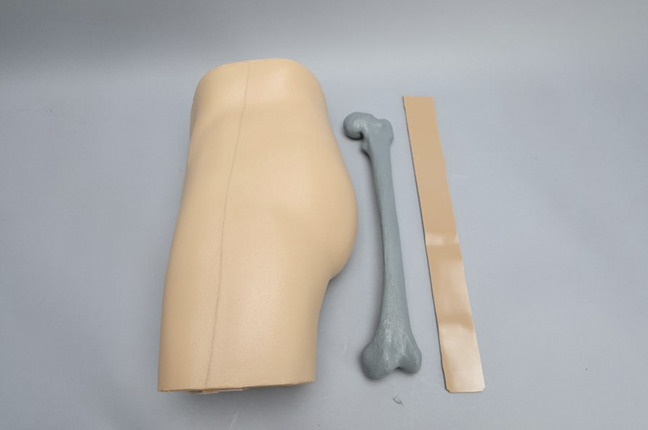
Photograph showing a customized phantom limb model (Sawbones) encasing a slipped capital femoral epiphysis Sawbones femur used to simulate a pediatric patient.

Twenty-one orthopaedic trainees, consisting of 9 junior orthopaedic residents and 12 senior medical students, were recruited from a single institution to participate in this study. A customized phantom limb model (Sawbones) encasing a SCFE radiopaque Sawbones femur was used to simulate a pediatric patient (Figure 1). Trainees were randomly assigned to one of the three educational platforms. After completing their respective educational platforms, each trainee was allowed one trial to perform in situ pin fixation on the phantom limb with conventional C-Arm (Cios Fusion; Siemens Sector Healthcare). Outcomes evaluated included overall surgical time, number of radiographs taken, Global Rating Scale (GRS) score, radiographic screw position, physical screw accuracy, presence of breaching of the articular surface or femoral neck, life-like rating of the physical module (0 to 10), and overall platform rating (0 to 10).

### Experimental Setup

**Figure 2:**
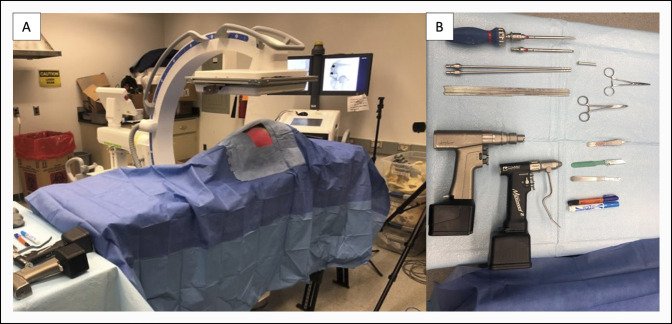
**A**, Photographs showing simulated operating room with C-arm, draping, and a slipped capital femoral epiphysis phantom model. **B**, Instrumentation setup.

**Figure 3:**
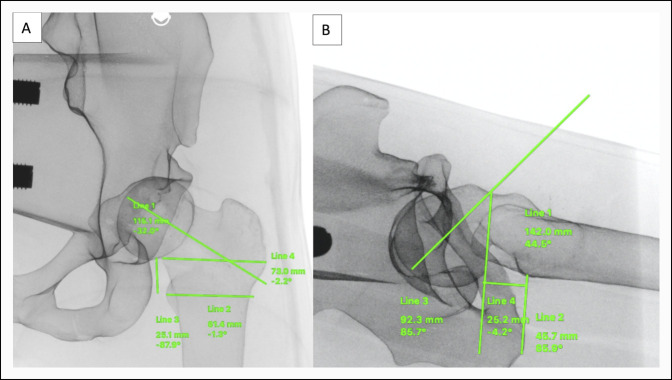
Radiographs showing surgical preoperative planning: participants were allowed to use preoperative planning software (Surgimap; Nemaris) to map out any planned screw trajectories on (**A**) AP and (**B**) lateral radiographs immediately before evaluation.

For each trial, a reusable phantom limb model of a left pediatric thigh was loaded with a grade III (severe) SCFE Sawbones model and placed on a radiolucent table. Bone models were replaced with a new sample between every trial. Before beginning a trial, a felt sticker was used to cover previous incisions sites on the reusable model. Surgical instruments included a scalpel, 2.8-mm Kirschner wire with power, a 4.5-mm drill bit with power, and a 7.0-mm cannulated screw (OrthoPediatrics). Operating room setup and instrument tray are shown in Figure 2. Trainees were given the opportunity to visualize AP and lateral preoperative radiographs of the SCFE femur to plan guide pin trajectory and start point using preoperative planning software (Surgimap; Nemaris) immediately before the trial (Figure 3).

### Training Platforms

**Figure 4:**
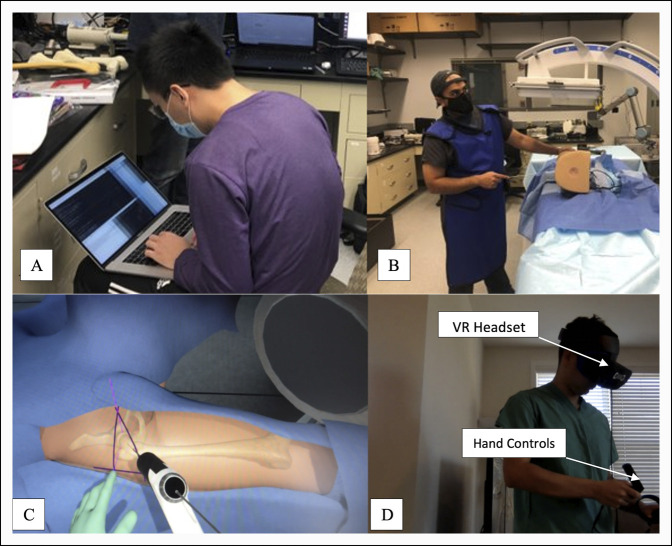
Photographs showing participants being randomized into three groups. **A**, Group 1: standardized reading or video material. **B**, Group 2: physical simulation with slipped capital femoral epiphysis model. **C**, Group 3: virtual reality simulation. **D**, Participant using virtual reality (VR) headset and hand controllers.

Trainees were assigned to one of the three educational platforms: (1) group 1 (control group): standardized reading and video material^10,11,12,13^ on how to perform in situ screw fixation (Appendix I, http://links.lww.com/JG9/A202), (2) group 2 (PS group): PS training on the SCFE Sawbones model, or (3) group 3 (VR group): VR-based SCFE simulation training module using the Osso VR platform (Figure 4). All groups had access to the standardized reading and video material. Trainees in group 1 were not allowed to use any resources or training material outside the ones that were provided. PS training consisted of a timed 15-minute session in which participants were allowed to practice guide pin placement on the SCFE Sawbones model. During this time, participants were allowed to ask any questions regarding SCFE screw fixation, interact with the physical model and surgical instruments, and practice the procedure. Participants in the VR group were trained on the Osso VR platform and asked to complete a standardized SCFE fixation module (Osso VR). This module allows users to freely manipulate the C-arm virtually from the AP to lateral positions during the procedure. The program also requires users to not only obtain images but also interpret them to manually adjust the position of the guide wire on the screen using physical hand controllers. Hand controllers did not provide any tactile feedback to users, with exception to vibration when using an instrument. Participants were asked to repeat the module until they achieved three “A” grades on pin placement accuracy, as graded within the VR module. Participants were not timed during completion of the VR practice modules.

### C-arm Imaging Settings

Trainees were given the option to either maintain the C-arm in a single position and manually maneuver the limb to obtain an AP and frog-leg lateral view or position the C-arm while keeping the phantom model static and obtaining an AP and lateral view. An imaging technology was available for all C-arm trials to assist in intraoperative positioning of the C-arm. Total number of AP and lateral images were recorded for each trial.

### Screw Physical Accuracy

**Figure 5:**
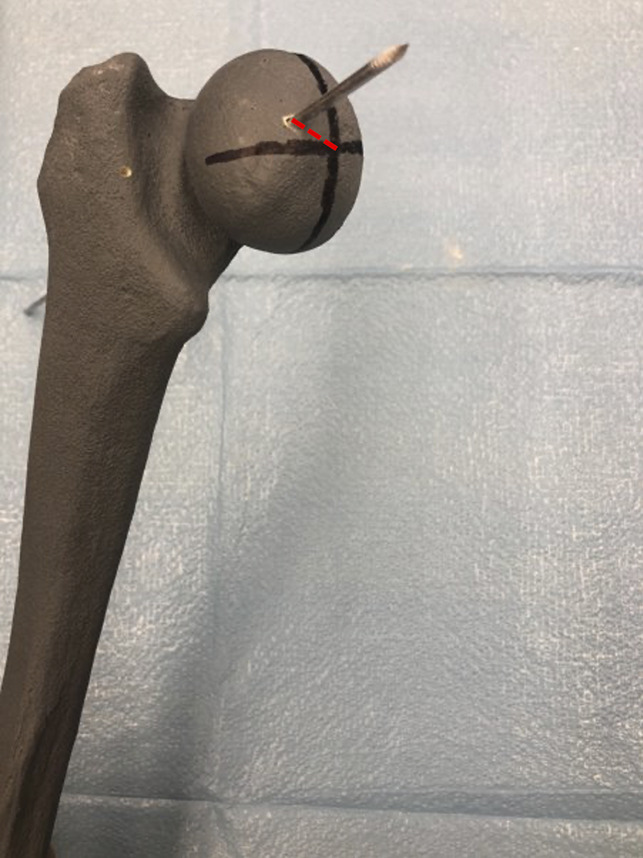
Photograph showing Kirschner wire in relation to the center of the femoral head. Physical accuracy was measured as the distance from the center of the hash mark to the Kirschner wire (dashed line).

To gauge physical accuracy, the center of the femoral head was marked preoperatively to indicate optimal screw trajectory. After placement of the screw, a 2.8-mm Kirschner wire was used to breech the cortex of the femoral head to assess postoperative screw trajectory. Screw placement accuracy was measured as the shortest distance from the preoperative marked site on the femoral head to actual postoperative trajectory (Figure 5).

### Radiographic Evaluation

**Figure 6:**
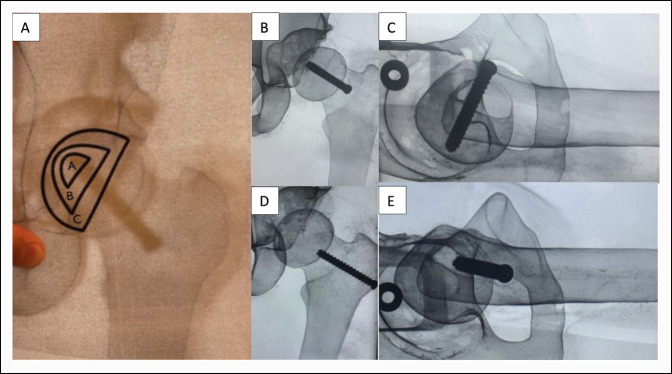
Illustration showing (**A**–**C**) grading system template of radiographic screw position as previously described by Pring et al. **A**, Radiograph template used to evaluate radiographic accuracy. **B**, AP view: example of a “grade A” screw. **C**, Lateral view: example of a “grade A” screw. **D**, AP view: example of a “grade C” screw. **E**, Lateral view: example of “grade C” screw.

Radiographic accuracy of pin placement within the epiphysis was graded on both AP and lateral radiographs using an A-C system as previously described by Pring et al.^14^ As described by Pring et al, “grade A” accuracy of pinning implied that the pin was placed within the central 50% of the physeal width, with the screw tip 5 to 10 mm across the physis, at least 5 mm from the subchondral bone, and at an angle of 70° to 90° to the capital physis. “Grade B” accuracy of pinning was reported when the pin was placed outside the “A” area, but not in the “C” area. This was considered to be an area containing the central 75% minus the central 50% of the physeal width, with the screw tip less than 5 mm across the physis or less than 5 mm from the subchondral bone, and at an angle of 50° to 90° to the physis. “Grade C” accuracy of pinning described screws placed outside the central 75% of the physeal width, less than 2.5 mm of screw tip across the physis, less than 2.5 mm from the subchondral bone, or at an angle less than 50° to the physis (Figure 6). Two board-certified orthopaedic surgeons (A.J. and B.H.) graded screw trajectory independently using the A-C grading system template.

### Surgical Performance Evaluation

In addition, both providers independently graded blinded recorded videos of participants on skill level using GRSs. GRSs have been developed as generic tools that assess performance across several skill domains (including respect for tissue, time, and motion, instrument handling/knowledge, flow of operation, knowledge of specific procedure, and communication skills) to generate an overall performance rating (range: 6 to 30 in our study). GRSs have been used in multiple surgical specialties in both simulated and clinical settings.^15^

### Articular Breaching and Femoral Neck In-out-in Assessment

**Figure 7:**
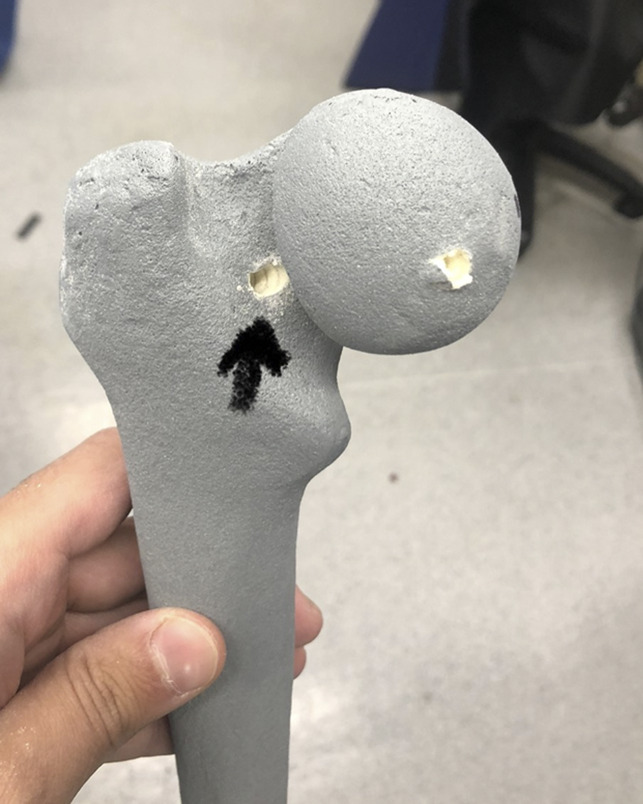
Photograph showing an arrow denoting femoral neck breeching posteriorly.

Rates of screw articular breaching and femoral neck in-out-in screw placement were recorded for each group. Articular breeching was defined as screw penetration through the articular surface of the femoral head. In-out-in was defined as screw penetration outside the femoral neck and re-entry into bone at a more proximal location within the femoral neck or head. Examples of screw breach and in-out-in screw placement are shown in Figure 7.

### Statistical Analyses

We used standard analysis of variance testing to compare continuous variables and chi-square test to compare categorical variables. Statistical significance was defined as a *P* value of 0.05 or less. Statistical analyses were done with Stata, version 15 (StataCorp LP).

## Results

### Study Participants

We recruited 21 junior orthopaedic residents and medical students from a single institution to participate in this study. Participants were equally divided into each of the three study platforms, with each group consisting of four medical students and three junior residents. All study participants completed the standardized reading and video study material before participation and verified that no additional learning tools were used.

### Objective Parameters

#### Screw Physical Accuracy

No significant differences were observed in mean (±SD) physical accuracy of the screw placement between all three study platforms (PS: 10.5 ± 7.9 mm, control: 9.4 ± 7.7 mm, VR: 13.3 ± 10.2 mm, *P* = 0.69).

#### Surgical Time and Amount of Radiographs Taken Intraoperatively

No significant differences were observed in mean (±SD) total surgical time between all three study platforms (PS: 14.2 ± 5.2 minutes, control: 24.4 ± 16.3 minutes, VR: 28.5 ± 10.2 minutes, *P* = 0.085). Compared with the control and PS groups, participants in the VR group took significantly greater numbers of AP, frog-leg/lateral, and total radiographs (Table 1). No differences were observed in the number of AP (*P* = 0.31), frog-leg/lateral (*P* = 0.83), and total radiographs (*P* = 0.62) between the PS or control groups.

**Table 1:** Mean ± SD of the Number of Radiographs Taken in Each Group

Factor	Physical Simulation	Control	Virtual Reality	*P* Value
AP radiographs	21.9 ± 7.4	28.9 ± 15.7	51.3 ± 20.9	0.007^a^
Lateral radiographs	21.7 ± 9.9	20.3 ± 14.6	53.1 ± 27.2	0.006^a^
Total radiographs	44.3 ± 8.1	49.1 ± 24.2	104.4 ± 46.3	0.003^a^

a Denotes significance.

#### Articular Breeching and Femoral Neck In-out-in Assessment

Across study platforms, no significant differences were observed in the number of participants who breached the articular surface of femoral head (PS: 5/7, control: 3/7, VR: 3/7, *P* = 0.47). No differences were also observed in the number of screws that breeched the femoral neck before entering the femoral head (PS: 0/7, control: 2/7, VR: 4/7, *P* = 0.061).

#### Radiographic Evaluation

**Figure 8:**
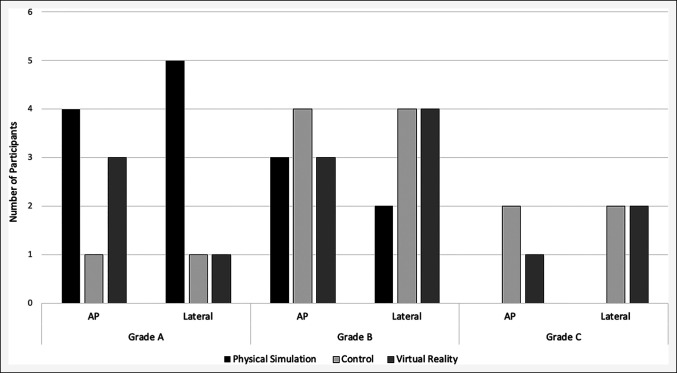
Graph showing radiographic grades in AP and lateral views across all three virtual reality platforms. As shown, physical simulation had the greatest number of grade A scores in both views. Virtual reality had more grade A scores than control only in the AP view.

When assessed by two independent reviewers (B.H. and A.J.), there were no significant differences in AP (Table 2) or lateral (Table 3) radiographic accuracy of screw placement across all three study platforms, as evaluated by the ABC grading system using templates overlayed onto the radiographs (AP: *P* = 0.413, lateral: *P* = 0.118, Figure 8). Inter-rater reliability was rated to be “perfect” (kappa = 1), with no disagreements.

**Table 2:** Anteroposterior Radiographic Grading of Screw Placement Accuracy Across Three Study Platforms

Grade	Physical Simulation	Control	Virtual Reality
A	4/7 (57%)	1/7 (14.3%)	3/7 (42.8%)
B	3/7 (43%)	4/7 (57%)	3/7 (42.8%)
C	0/7 (0%)	2/7 (28.5%)	1/7 (14.3%)
Chi-square	*P* = 0.413		

^a^Denotes significance.

**Table 3:** Lateral Radiographic Grading of Screw Placement Accuracy Across Three Study Platforms

Grade	Physical Simulation	Control	Virtual Reality
A	5/7 (71%)	1/7 (14.3%)	1/7 (14.3%)
B	2/7 (28.5%)	4/7 (57%)	4/7 (57%)
C	0/7 (0%)	2/7 (28.5%)	2/7 (28.5%)
Chi-square	*P* = 0.118		

#### Surgical Performance Evaluation

Across all three study platforms, no significant differences were observed in GRS scores (reviewer 1: *P* = 0.11, reviewer 2: *P* = 0.06; Table 4). Paired *t*-test analysis for each participant determined no significant differences in GRS scores between the two reviewers for a given participant (reviewer 1: 15.3, reviewer 2: 15.24, *P* = 0.74).

**Table 4:** Surgical Evaluation Scores (Mean ± SD) Across Three Study Platforms by Two Independent Reviewers

Factor	Physical Simulation	Control	Virtual Reality	*P* Value
Reviewer 1	19 ± 7.5	14.3 ± 5.3	12.7 ± 2.6	0.11
Reviewer 2	19.1 ± 7.0	13.8 ± 4.6	12.7 ± 2.2	0.06

### Subjective Participant Evaluation

#### Life-like Score

When participants were asked to evaluate how life-like (Likert scale: 0 to 10, 0 = not life-like, 10 = extremely life-like) their study platform was, no significant differences were observed in mean (±SD) between the three groups (PS: 8 ± 2, control: 7.7 ± 1.4, VR: 7 ± 3.2, *P* = 0.71).

#### Utility Score

Participants ranked PS as the most useful study platform compared with the control group and VR group (PS: 9.7 ± 0.5, control: 6.0 ± 1.0, VR: 7.3 ± 2.3, *P* = 0.0006).

#### Comments

Participants in the PS group reported that the study platform was “extremely useful,” “felt like an operating room,” and “was an extremely enjoyable experience.” Participants in the control group reported that the “videos were excellent and useful” but reported that “having a physical model would have been more helpful.” Participants in the VR group believed that “it was helpful in understanding concepts of surgery” and “useful for the portion of the physical simulation when inserting the Kirschner wire.”

## Discussion

We conducted a prospective study comparing the educational utility of VR to other learning methods in improving the surgical performance of orthopaedic trainees for in situ screw fixation of high-grade SCFE. We assessed objective parameters, including screw accuracy, breaching of the femoral head or neck, surgical time, radiographic accuracy, surgical technique, and collected subjective evaluations of each educational platform. Our results indicate that no significant differences were observed in most objective parameters. Subjectively, participants preferred PS, followed by VR, and then conventional book/video material. Overall, our results may suggest that although practicing with a physical SCFE model may be the most realistic method to train novice surgeons, VR training modules may yield similar radiographic accuracy and surgical technique performance while offering a more convenient to practice surgical skills.

VR simulation has been previously described to improve resident skill level in the operating room or on cadaver models for knee arthroscopy, shoulder arthroscopy, and total hip arthroplasty.^1,2,3,4,5,6^ For example, a randomized controlled trial by Banaszek et al^1^ found that residents who trained with a knee arthroscopy VR simulator that provided haptic and tactile feedback had superior GRS scores in a cadaver model compared with residents who trained with a bench-top Sawbones model. More modern VR simulators with head-mounted displays have also demonstrated improvements in surgical performance, even among novice trainees. For example, a randomized trial by Blumstein et al demonstrated that compared with passive methods of learning (technique guide review), VR headsets with hand controllers provided improved procedural in intramedullary nailing among first-year and second-year medical students. However, educational benefits of modern VR surgical training platforms and the transferability of skills to a physical model have never been evaluated in pediatric orthopaedic procedures.

Unlike the performance benefits previously reported in intramedullary nailing procedures among novices, our results indicated that no notable differences were observed in most objective parameters (screw accuracy, radiographic grade, surgical technique, and breaching) between a modern VR headset and practice with a physical model. These results are likely secondary to (1) the relative technical difficulty of in situ SCFE fixation and (2) the lack of notable prior experience with SCFE fixation in our cohort of participants. SCFE fixation with in situ screws or pins has been previously associated with high rates of complications, even when done by experienced providers. For example, in a cohort of 202 pediatric patients with SCFE treated with screw fixation by trained orthopaedic surgeons, Riley et al^16^ reported a complication rate of 26%, with joint penetration being the most common complication. Therefore, the inherent difficulty of performing this procedure may have masked any short-term gains in surgical performance from VR training in an extremely novice cohort. Moreover, the learning curve for successfully performing a SCFE procedure likely requires more than one trial. Although the minimum number of procedures to gain proficiency in SCFE fixation has not been previously quantified, the recommended number of supervised practice procedures for pediatric orthopaedic fellows for other procedures, such as supracondylar humerus pin fixation, has been reported to be approximately 15.^17^ Therefore, in our novice cohort, increased exposure to the basics of SCFE fixation and multiple trials may have resulted in greater differences among various educational platforms.

Although our results indicated that VR training offered similar performance as a phantom limb or a didactic teaching model, the mobility of modern VR platforms may offer extra convenience benefits. The novel coronavirus pandemic in 2020 forced orthopaedic residency curriculums to develop and adopt more remote-based learning modalities.^18^ Often times, these include more didactic sessions and video/text-based learning modules to decrease resident exposure to the virus.^18^ VR training modules may offer excellent supplementary content to didactic learning because it enables residents to directly visualize important surgical concepts and repeat procedures multiple times from the comfort and safety of their home while avoiding skill decay. However, despite the convenience of this system, program directors must consider the cost of the Osso VR platform. The hardware alone needed to run the Osso VR module is approximately 300 dollars for a single unit; residency leadership may consider bulk ordering the hardware and software implants from Osso for resident access as part of institutional educational funds. Although training with cadaver models may offer a more cost-effective method of instruction, because these specimens can be bulk ordered and used for multiple procedures across many residents, access to these models during times of social distancing and mandatory remote learning may preclude residents from participating in such learning opportunities. In this scenario specifically, surgical education with VR technology may be particularly useful and have a justified upfront cost.

Although the portability of the VR headset system offers notable convenience and advantages during a pandemic, participants offered some suggestions for VR training improvement to better suit their needs. First, the controllers with the VR system are not modular. As such, the same hand controller must represent a variety of different surgical instruments, decreasing the realism of the virtual simulation and potentially limiting gains in actual surgical technique. This could be rectified with addition of secondary controllers or instruments in the same VR kit. Second, we did not use the available test mode in the VR module, which would allow the user to critically think about pin placement rather than following outlined trajectories in the training module. Users may have gained further improvements in performance if both training and testing modules were completed before surgical simulation. Finally, the VR headset module for SCFE fixation does not have any modality to artificially palpate anatomic landmarks on the extremity to mark the incision site or feel differences in resistance while placing the guide wire. Although tactile components of anatomy are primarily limited by the portability of the system, ability to identify the incision site should be tested in the VR modules. These missing attributes in VR learning are key to mastering SCFE fixation and highlight the benefits of using a physical or cadaver model. Moreover, these limitations may explain why the VR group required greater radiograph images during the evaluation process.

There are limitations to our study. First, our study had a small sample size, reducing the external validity of our results. Owing to the low sample size, outliers in the data set may have more notable effects on the mean. For example, one participant in the VR group took a total 185 radiographs during surgical simulation, compared with the next highest participant in the group with 132 radiographs. Notably, although there were no statistically significant differences in outcomes between all three platforms, our findings seem to trend toward favoring PS training over VR. Perhaps with a greater study sample size, these differences may be further pronounced. Moreover, individuals in the PS group were allowed to train and subsequently evaluated on the same phantom limb, which likely introduced some bias. Finally, using a sample of participants with limited orthopaedic experience may result in diminished gains in improvement with any educational platform. Participants may require prolonged exposure to the physical model and fundamentals of SCFE fixation to realize benefits from any particular surgical training methodology.

## Conclusion

VR training holds promise for the future of orthopaedic training, and no statistically significant differences were observed in in situ screw placement accuracy, radiographic grades, and surgical technique compared with practice with a physical model in novice orthopaedic trainees. However, those trained with physical models performed nominally better across most of the outcomes and subjectively evaluated this method of training as the most life-like and useful. Future work may focus on improving current VR technology to make controllers more modular and realistic while simultaneously expanding training modules to be more detailed-oriented and rigorous (http://links.lww.com/JG9/A203 and http://links.lww.com/JG9/A204).

## References

[1] Banaszek D You D Chang J : Virtual reality compared with bench-top simulation in the acquisition of arthroscopic skill: A randomized controlled trial. J Bone Joint Surg Am 2017;99:e34. 28375898 10.2106/JBJS.16.00324

[2] Camp CL Krych AJ Stuart MJ Regnier TD Mills KM Turner NS: Improving resident performance in knee arthroscopy: A prospective value assessment of simulators and cadaveric skills laboratories. J Bone Joint Surg Am 2016;98:220-225. 26842412 10.2106/JBJS.O.00440

[3] Cannon WD Garrett WE Hunter RE : Improving residency training in arthroscopic knee surgery with use of a virtual-reality simulator: A randomized blinded study. J Bone Joint Surg Am 2014;96:1798-1806. 25378507 10.2106/JBJS.N.00058

[4] Logishetty K Rudran B Cobb JP: Virtual reality training improves trainee performance in total hip arthroplasty: A randomized controlled trial. Bone Joint J 2019;101-B:1585-1592. 31786991 10.1302/0301-620X.101B12.BJJ-2019-0643.R1

[5] Rebolledo BJ Hammann-Scala J Leali A Ranawat AS: Arthroscopy skills development with a surgical simulator: A comparative study in orthopaedic surgery residents. Am J Sports Med 2015;43:1526-1529. 25769535 10.1177/0363546515574064

[6] Waterman BR Martin KD Cameron KL Owens BD Belmont PJ: Simulation training improves surgical proficiency and safety during diagnostic shoulder arthroscopy performed by residents. Orthopedics 2016;39:e479-e485. 27135460 10.3928/01477447-20160427-02

[7] Bartlett JD Lawrence JE Stewart ME Nakano N Khanduja V: Does virtual reality simulation have a role in training trauma and orthopaedic surgeons? Bone Joint J 2018;100-B:559-565. 29701089 10.1302/0301-620X.100B5.BJJ-2017-1439

[8] Blumstein G Zukotynski B Cevallos N : Randomized trial of a virtual reality tool to teach surgical technique for tibial shaft fracture intramedullary nailing. J Surg Educ 2020;77:969-977. 32035854 10.1016/j.jsurg.2020.01.002 PMC7351249

[9] Orland MD Patetta MJ Wieser M Kayupov E Gonzalez MH: Does virtual reality improve procedural completion and accuracy in an intramedullary tibial nail procedure? A randomized control trial. Clin Orthop Relat Res 2020;478:2170-2177. 32769533 10.1097/CORR.0000000000001362 PMC7431248

[10] OrthoPediatrics: 7.0 mm Cannulated Screw. Warsaw, IN, OrthoPediatrics, 2020.

[11] Davidson RS Caird MS: Percutaneous in situ cannulated screw fixation of the slipped capital femoral epiphysis. Oper Tech Orthop Pediatr Surg 2012:564-569.

[12] Sankar WN: In situ pinning of a stable SCFE: How I do it? POSNA Acad 2017. http://www.posnacademy.org/media/In+Situ+Pinning+of+a+Stable+SCFE/0_c2ok07sb/19140172. Accessed October 16, 2020.

[13] Loder RT: Author's preferred technique: How to pin a SCFE. POSNA Acad 2014. https://www.posnacademy.org/media/How+to+Pin+a+SCFE/0_stjvaeph. Accessed October 16, 2020.

[14] Pring ME Adamczyk M Hosalkar HS Bastrom TP Wallace CD Newton PO: In situ screw fixation of slipped capital femoral epiphysis with a novel approach: A double-cohort controlled study. J Child Orthop 2010;4:239-244.

[15] Niitsu H Hirabayashi N Yoshimitsu M : Using the Objective Structured Assessment of Technical Skills (OSATS) global rating scale to evaluate the skills of surgical trainees in the operating room. Surg Today 2013;43:271-275. 22941345 10.1007/s00595-012-0313-7 PMC3574562

[16] Riley PM Weiner DS Gillespie R Weiner SD: Hazards of internal fixation in the treatment of slipped capital femoral epiphysis. J Bone Joint Surg Am 1990;72:1500-1509. 2254358

[17] Liu RW Roocroft J Bastrom T Yaszay B: Surgeon learning curve for pediatric supracondylar humerus fractures. J Pediatr Orthop 2011;31:818-824. 22101658 10.1097/BPO.0b013e3182306884

[18] Sabharwal S Ficke JR LaPorte DM: How we do it: Modified residency programming and adoption of remote didactic curriculum during the COVID-19 pandemic. J Surg Educ 2020;77:1033-1036. 32546387 10.1016/j.jsurg.2020.05.026 PMC7253931

